# Diethyl 2,5-bis­[(1*E*)-(1*H*-pyrrol-2-yl­methyl­idene)amino]­thio­phene-3,4-dicarboxyl­ate

**DOI:** 10.1107/S1600536811031576

**Published:** 2011-08-11

**Authors:** Stéphane Dufresne, W. G. Skene

**Affiliations:** a Department of Chemistry, University of Montreal, CP 6128, succ. Centre-ville, Montréal, Québec, Canada H3C 3J7

## Abstract

In the crystal structure of the title compound, C_20_H_20_N_4_O_4_S, the azomethine group adopt *E* conformations. The pyrrole units are twisted by 10.31 (4) and 18.90 (5)° with respect to the central thio­phene ring. The three-dimensional network is close packed and involves N—H⋯O, N—H⋯N, C—H⋯N and C—H⋯O hydrogen bonding.

## Related literature

For general background, see: Dufresne *et al.* (2007 ▶, 2011 ▶). For thio­phene azomethines, see: Dufresne *et al.* (2006 ▶, 2010*a* ▶,*b* ▶). For alkene comparison, see: Ruban *et al.* (1975 ▶); Zobel *et al.* (1978 ▶).
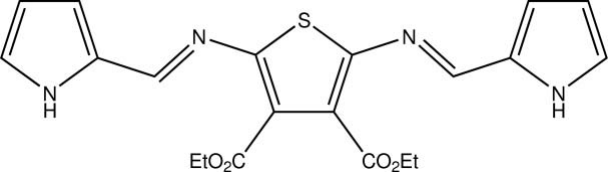


## Experimental

### 

#### Crystal data

C_20_H_20_N_4_O_4_S
*M* *_r_* = 412.46
Orthorhombic, 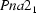

*a* = 16.898 (3) Å
*b* = 12.643 (3) Å
*c* = 9.4220 (19) Å
*V* = 2012.9 (7) Å^3^
*Z* = 4
Cu *K*α radiation
μ = 1.73 mm^−1^
*T* = 150 K
0.10 × 0.03 × 0.03 mm

#### Data collection

Bruker SMART 6000 diffractometer
Absorption correction: multi-scan (*SADABS*; Sheldrick, 1996 ▶) *T*_min_ = 0.841, *T*_max_ = 0.947
17313 measured reflections
3040 independent reflections
3004 reflections with *I* > 2σ(*I*)
*R*_int_ = 0.028

#### Refinement

*R*[*F* ^2^ > 2σ(*F* ^2^)] = 0.024
*wR*(*F* ^2^) = 0.064
*S* = 1.04
3040 reflections
264 parameters
1 restraint
H-atom parameters constrained
Δρ_max_ = 0.19 e Å^−3^
Δρ_min_ = −0.15 e Å^−3^
Absolute structure: Flack (1983) ▶, 1275 Friedel pairs
Flack parameter: 0.085 (12)

### 

Data collection: *SMART* (Bruker, 2003 ▶); cell refinement: *SAINT* (Bruker, 2004 ▶); data reduction: *SAINT*; program(s) used to solve structure: *SHELXS97* (Sheldrick, 2008 ▶); program(s) used to refine structure: *SHELXL97* (Sheldrick, 2008 ▶); molecular graphics: *SHELXTL* (Sheldrick, 2008 ▶) and *ORTEP-3* (Farrugia, 1997 ▶); software used to prepare material for publication: *UdMX* (Marris, 2004 ▶).

## Supplementary Material

Crystal structure: contains datablock(s) I, global. DOI: 10.1107/S1600536811031576/zq2118sup1.cif

Structure factors: contains datablock(s) I. DOI: 10.1107/S1600536811031576/zq2118Isup2.hkl

Supplementary material file. DOI: 10.1107/S1600536811031576/zq2118Isup3.cml

Additional supplementary materials:  crystallographic information; 3D view; checkCIF report

## Figures and Tables

**Table 1:** Hydrogen-bond geometry (Å, °)

*D*—H⋯*A*	*D*—H	H⋯*A*	*D*⋯*A*	*D*—H⋯*A*
N1—H100⋯O3^i^	0.88	2.37	3.040 (2)	133
N4—H400⋯O1^ii^	0.88	2.47	3.174 (2)	138
N4—H400⋯N2^ii^	0.88	2.59	3.2136 (19)	128
C3—H3⋯N4^iii^	0.95	2.56	3.441 (2)	155
C13—H13⋯O3^iv^	0.95	2.51	3.126 (2)	123

Symmetry codes: (i) 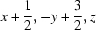; (ii) 
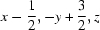
; (iii) 
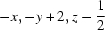
; (iv) 
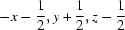
.

**Fig. 1.**
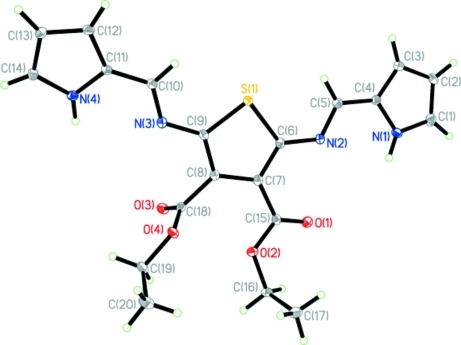
The molecular structure of the title compound with thermal ellipsoids drawn at 30% probability level.

**Fig. 2.**
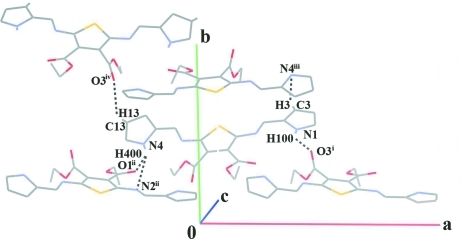
Supramolecular structure showing the intermolecular hydrogen bonding (dashed lines). Symmetry codes: (i) x+1/2, -y+3/2, z; (ii) x-1/2, -y+3/2, z; (iii) -x, -y+2, z-1/2; (iv) -x-1/2, y+1/2, z-1/2.

## supplementary crystallographic information

### Comment

During our on-going research relating to conjugated azomethines (Dufresne *et al.*, 2007; Dufresne & Skene, 2010*a*,*b*; Dufresne & Skene, 2011) we prepared the title compound (I), C_20_H_20_N_4_O_4_S. To the best of our knowledge, there are very few reported crystal structures of azomethines consisting of pyrrole and thiophene units together. The molecular structure was confirmed by a X-ray diffraction study (Fig. 1). Neither solvent molecules nor counter-ions were found in the closed-packing of the crystal structure.

A major point of interest is the azomethine bond which adopts the *E* configuration. The bond lengths for C4—C5, N2—C5 and N2—C6 are 1.429 (3), 1.294 (3) and 1.379 (2) Å, respectively. The related bonds C10—C11, N3—C10 and N3—C9 are 1.434 (3), 1.288 (3) and 1.379 (3) Å, respectively. All bond distances are consistent with those of a similar conjugated compound consisting uniquely of thiophenes with two azomethine bonds (Dufresne *et al.*, 2006). The analogues bond lengths for the all-thiophene counterpart are: 1.441 (4), 1.272 (3) and 1.388 (3) Å. It was found that the three heterocycles of (I) are not perfectly coplanar. This was confirmed by measuring the dihedral angles between the planes described by both terminal pyrroles and the plane described by the central thiophene. The dihedral angle between the N1-pyrrole and thiophene planes is 10.31 (4)°, while that for the thiophene and N4-pyrrole planes is 18.90 (5)°. The measured angles are similar to the all-thiophene analogue whose terminal thiophenes are twisted by 9.04 (4)° and 25.07 (6)° with the central thiophene. The pyrrole N-H is involved in several N—H···O and N—H···N donor-acceptor interactions while C—H···N and C—H···O are also observed (Table 1). All these interactions are responsible for the overall extended three-dimensional crystal network (Fig. 2), while no π-stacking was found.

### Experimental

In anhydrous toluene (25 mL) was added 1H-pyrrole-2-carbaldehyde to which was subsequently added DABCO, TiCl_4_ in toluene at 0 °C and then diethyl 2,5-diaminothiophene-3,4-dicarboxylate was added. The mixture was then refluxed for 30 minutes and the solvent, after which the solvent was removed. Purification by flash chromatography yielded the title product as a red solid. Single crystals were obtained by slow evaporation of an acetone solution of the title compound.

### Refinement

H atoms were placed in calculated positions and included in the refinement in the riding-model approximation, with C—H = 0.95 Å for aromatic H atoms, C—H = 0.99 Å for methylene H atoms, C—H = 0.98 Å for methyl H atoms, and *U*_iso_(H) = 1.2 *U*_eq_(C). The protons on the N atoms of the pyrrole groups were placed in calculated positions with N—H = 0.85 Å and included in the refinement in the riding-model approximation, with *U*_iso_(H) = 1.5 *U*_eq_(N).

### Crystal data

**Table:**

C_20_H_20_N_4_O_4_S	*D*_x_ = 1.361 Mg m^−^^3^
*M**_r_* = 412.46	Melting point: 483(2) K
Orthorhombic, *P**n**a*2_1_	Cu *K*α radiation, λ = 1.54178 Å
Hall symbol: P 2c -2n	Cell parameters from 4515 reflections
*a* = 16.898 (3) Å	θ = 4.4–70.6°
*b* = 12.643 (3) Å	µ = 1.73 mm^−^^1^
*c* = 9.4220 (19) Å	*T* = 150 K
*V* = 2012.9 (7) Å^3^	Block, orange
*Z* = 4	0.10 × 0.03 × 0.03 mm
*F*(000) = 864	

### Data collection

**Table:**

Bruker SMART 6000 diffractometer	3040 independent reflections
Radiation source: Rotating Anode	3004 reflections with *I* > 2σ(*I*)
Montel 200 optics	*R*_int_ = 0.028
Detector resolution: 5.5 pixels mm^-1^	θ_max_ = 66.6°, θ_min_ = 4.4°
ω scans	*h* = −19→19
Absorption correction: multi-scan (*SADABS*; Sheldrick, 1996)	*k* = −14→14
*T*_min_ = 0.841, *T*_max_ = 0.947	*l* = −9→10
17313 measured reflections	

### Refinement

**Table:**

Refinement on *F*^2^	Secondary atom site location: difference Fourier map
Least-squares matrix: full	Hydrogen site location: inferred from neighbouring sites
*R*[*F*^2^ > 2σ(*F*^2^)] = 0.024	H-atom parameters constrained
*wR*(*F*^2^) = 0.064	*w* = 1/[σ^2^(*F*_o_^2^) + (0.0475*P*)^2^ + 0.2939*P*] where *P* = (*F*_o_^2^ + 2*F*_c_^2^)/3
*S* = 1.04	(Δ/σ)_max_ < 0.001
3040 reflections	Δρ_max_ = 0.19 e Å^−^^3^
264 parameters	Δρ_min_ = −0.15 e Å^−^^3^
1 restraint	Absolute structure: Flack (1983), 1275 Friedel pairs
Primary atom site location: structure-invariant direct methods	Flack parameter: 0.085 (12)

### Special details

**Table:**

Geometry. All esds (except the esd in the dihedral angle between two l.s. planes) are estimated using the full covariance matrix. The cell esds are taken into account individually in the estimation of esds in distances, angles and torsion angles; correlations between esds in cell parameters are only used when they are defined by crystal symmetry. An approximate (isotropic) treatment of cell esds is used for estimating esds involving l.s. planes.
Refinement. Refinement of F^2^ against ALL reflections. The weighted R-factor wR and goodness of fit S are based on F^2^, conventional R-factors R are based on F, with F set to zero for negative F^2^. The threshold expression of F^2^ > 2sigma(F^2^) is used only for calculating R-factors(gt) etc. and is not relevant to the choice of reflections for refinement. R-factors based on F^2^ are statistically about twice as large as those based on F, and R- factors based on ALL data will be even larger.

### Fractional atomic coordinates and isotropic or equivalent isotropic displacement parameters (Å^2^)

**Table:**

	*x*	*y*	*z*	*U*_iso_*/*U*_eq_	
S1	0.025121 (19)	0.93934 (3)	0.61244 (5)	0.01883 (11)	
O1	0.19929 (6)	0.77328 (9)	0.93257 (16)	0.0238 (3)	
O2	0.08597 (7)	0.76090 (9)	1.05985 (15)	0.0233 (3)	
O3	−0.07216 (7)	0.66172 (8)	0.89456 (15)	0.0223 (3)	
O4	−0.08363 (6)	0.80055 (8)	1.04346 (14)	0.0199 (3)	
N1	0.34790 (7)	0.92360 (10)	0.62911 (19)	0.0211 (3)	
H100	0.3404	0.8921	0.7113	0.025*	
N2	0.18309 (7)	0.90938 (10)	0.69168 (16)	0.0176 (3)	
N3	−0.12532 (7)	0.86607 (10)	0.69517 (18)	0.0186 (3)	
N4	−0.29025 (7)	0.83824 (9)	0.63130 (17)	0.0176 (3)	
H400	−0.2741	0.7876	0.6885	0.021*	
C1	0.41958 (10)	0.94223 (13)	0.5679 (2)	0.0274 (5)	
H1	0.4695	0.9235	0.6069	0.033*	
C2	0.40779 (9)	0.99277 (13)	0.4398 (2)	0.0255 (4)	
H2	0.4476	1.0149	0.3751	0.031*	
C3	0.32546 (9)	1.00526 (12)	0.4233 (2)	0.0237 (4)	
H3	0.2996	1.0376	0.3449	0.028*	
C4	0.28871 (9)	0.96205 (12)	0.54149 (19)	0.0180 (4)	
C5	0.20611 (9)	0.95575 (11)	0.5762 (2)	0.0188 (4)	
H5	0.1680	0.9858	0.5139	0.023*	
C6	0.10367 (9)	0.89476 (11)	0.72176 (19)	0.0171 (3)	
C7	0.07559 (9)	0.83597 (11)	0.8356 (2)	0.0173 (3)	
C8	−0.00907 (9)	0.82379 (11)	0.83286 (19)	0.0163 (3)	
C9	−0.04488 (9)	0.87299 (11)	0.7205 (2)	0.0177 (3)	
C10	−0.15923 (9)	0.92361 (11)	0.5995 (2)	0.0177 (3)	
H10	−0.1285	0.9729	0.5471	0.021*	
C11	−0.24267 (9)	0.91463 (12)	0.57050 (19)	0.0166 (3)	
C12	−0.29035 (9)	0.97881 (12)	0.4860 (2)	0.0197 (3)	
H12	−0.2734	1.0374	0.4307	0.024*	
C13	−0.36866 (10)	0.94050 (11)	0.4980 (2)	0.0206 (4)	
H13	−0.4142	0.9688	0.4526	0.025*	
C14	−0.36664 (8)	0.85413 (11)	0.5881 (2)	0.0200 (4)	
H14	−0.4110	0.8127	0.6154	0.024*	
C15	0.12846 (9)	0.78771 (11)	0.9451 (2)	0.0177 (4)	
C16	0.12941 (10)	0.71688 (13)	1.1806 (2)	0.0245 (4)	
H16A	0.1752	0.6754	1.1459	0.029*	
H16B	0.0945	0.6688	1.2350	0.029*	
C17	0.15813 (11)	0.80532 (14)	1.2762 (2)	0.0294 (4)	
H17A	0.1958	0.8497	1.2243	0.044*	
H17B	0.1842	0.7751	1.3599	0.044*	
H17C	0.1130	0.8484	1.3064	0.044*	
C18	−0.05767 (8)	0.75280 (11)	0.9270 (2)	0.0168 (3)	
C19	−0.12474 (10)	0.73187 (13)	1.1459 (2)	0.0250 (4)	
H19A	−0.0956	0.6645	1.1578	0.030*	
H19B	−0.1789	0.7156	1.1120	0.030*	
C20	−0.12819 (12)	0.79079 (15)	1.2846 (2)	0.0325 (4)	
H20A	−0.0744	0.8084	1.3153	0.049*	
H20B	−0.1536	0.7463	1.3565	0.049*	
H20C	−0.1588	0.8560	1.2722	0.049*	

### Atomic displacement parameters (Å^2^)

**Table:**

	*U*^11^	*U*^22^	*U*^33^	*U*^12^	*U*^13^	*U*^23^
S1	0.01600 (17)	0.02159 (18)	0.0189 (3)	−0.00165 (12)	−0.00024 (17)	0.00547 (15)
O1	0.0191 (6)	0.0282 (6)	0.0243 (8)	0.0010 (4)	−0.0022 (5)	0.0040 (5)
O2	0.0212 (5)	0.0307 (6)	0.0179 (8)	0.0002 (5)	−0.0019 (5)	0.0047 (5)
O3	0.0294 (6)	0.0174 (5)	0.0201 (8)	−0.0038 (4)	0.0021 (5)	−0.0007 (4)
O4	0.0224 (5)	0.0197 (5)	0.0175 (8)	−0.0038 (4)	0.0047 (4)	−0.0006 (4)
N1	0.0192 (6)	0.0238 (6)	0.0204 (10)	0.0005 (5)	0.0019 (6)	0.0054 (6)
N2	0.0163 (6)	0.0179 (6)	0.0186 (10)	−0.0028 (5)	−0.0004 (5)	0.0008 (6)
N3	0.0166 (6)	0.0193 (6)	0.0199 (9)	−0.0007 (5)	0.0006 (5)	−0.0005 (5)
N4	0.0194 (6)	0.0160 (6)	0.0174 (9)	0.0025 (4)	−0.0001 (5)	0.0019 (5)
C1	0.0161 (8)	0.0342 (9)	0.0318 (14)	0.0000 (6)	0.0017 (7)	−0.0003 (8)
C2	0.0209 (8)	0.0331 (9)	0.0226 (12)	−0.0056 (6)	0.0054 (7)	0.0051 (7)
C3	0.0252 (8)	0.0249 (8)	0.0210 (12)	−0.0031 (6)	−0.0020 (7)	0.0045 (7)
C4	0.0172 (8)	0.0167 (7)	0.0201 (11)	−0.0009 (6)	−0.0019 (6)	0.0008 (6)
C5	0.0207 (8)	0.0151 (6)	0.0206 (12)	0.0003 (6)	−0.0008 (6)	0.0004 (6)
C6	0.0164 (8)	0.0145 (7)	0.0202 (10)	−0.0005 (5)	−0.0006 (6)	−0.0022 (6)
C7	0.0183 (8)	0.0142 (7)	0.0193 (10)	−0.0017 (5)	0.0001 (6)	−0.0038 (6)
C8	0.0174 (7)	0.0132 (7)	0.0183 (11)	−0.0003 (5)	0.0007 (6)	−0.0025 (6)
C9	0.0184 (7)	0.0162 (7)	0.0186 (10)	−0.0018 (5)	0.0025 (6)	0.0006 (6)
C10	0.0187 (7)	0.0167 (6)	0.0178 (11)	0.0009 (5)	0.0020 (7)	0.0012 (7)
C11	0.0195 (7)	0.0168 (7)	0.0136 (10)	0.0016 (6)	0.0027 (6)	−0.0008 (5)
C12	0.0220 (8)	0.0181 (7)	0.0190 (11)	0.0017 (6)	0.0007 (6)	0.0041 (6)
C13	0.0202 (8)	0.0196 (7)	0.0220 (11)	0.0030 (6)	−0.0027 (7)	0.0000 (7)
C14	0.0183 (7)	0.0185 (7)	0.0233 (12)	−0.0010 (5)	0.0001 (7)	−0.0011 (7)
C15	0.0191 (8)	0.0148 (7)	0.0190 (11)	−0.0022 (6)	0.0008 (7)	−0.0027 (6)
C16	0.0282 (9)	0.0274 (8)	0.0178 (11)	−0.0007 (6)	−0.0042 (7)	0.0061 (7)
C17	0.0335 (10)	0.0330 (9)	0.0215 (12)	−0.0004 (7)	−0.0028 (8)	0.0002 (8)
C18	0.0143 (7)	0.0174 (7)	0.0187 (11)	0.0008 (5)	−0.0017 (6)	0.0015 (7)
C19	0.0262 (8)	0.0271 (8)	0.0218 (13)	−0.0062 (6)	0.0072 (7)	0.0015 (7)
C20	0.0424 (10)	0.0323 (9)	0.0228 (13)	−0.0036 (7)	0.0076 (9)	−0.0003 (8)

### Geometric parameters (Å, °)

**Table:**

S1—C9	1.7717 (17)	C6—C7	1.389 (2)
S1—C6	1.7721 (17)	C7—C8	1.439 (2)
O1—C15	1.2165 (19)	C7—C15	1.495 (2)
O2—C15	1.342 (2)	C8—C9	1.369 (2)
O2—C16	1.464 (2)	C8—C18	1.506 (2)
O3—C18	1.2164 (19)	C10—C11	1.441 (2)
O4—C18	1.327 (2)	C10—H10	0.9500
O4—C19	1.472 (2)	C11—C12	1.393 (2)
N1—C1	1.362 (2)	C12—C13	1.414 (2)
N1—C4	1.385 (2)	C12—H12	0.9500
N1—H100	0.8800	C13—C14	1.384 (2)
N2—C5	1.296 (2)	C13—H13	0.9500
N2—C6	1.384 (2)	C14—H14	0.9500
N3—C10	1.292 (2)	C16—C17	1.516 (3)
N3—C9	1.383 (2)	C16—H16A	0.9900
N4—C14	1.369 (2)	C16—H16B	0.9900
N4—C11	1.381 (2)	C17—H17A	0.9800
N4—H400	0.8800	C17—H17B	0.9800
C1—C2	1.380 (3)	C17—H17C	0.9800
C1—H1	0.9500	C19—C20	1.505 (3)
C2—C3	1.409 (2)	C19—H19A	0.9900
C2—H2	0.9500	C19—H19B	0.9900
C3—C4	1.387 (3)	C20—H20A	0.9800
C3—H3	0.9500	C20—H20B	0.9800
C4—C5	1.436 (2)	C20—H20C	0.9800
C5—H5	0.9500		
			
C9—S1—C6	90.89 (8)	N4—C11—C10	123.11 (14)
C15—O2—C16	117.02 (13)	C12—C11—C10	128.93 (14)
C18—O4—C19	115.44 (12)	C11—C12—C13	107.25 (14)
C1—N1—C4	109.20 (16)	C11—C12—H12	126.4
C1—N1—H100	125.4	C13—C12—H12	126.4
C4—N1—H100	125.4	C14—C13—C12	107.22 (14)
C5—N2—C6	121.56 (15)	C14—C13—H13	126.4
C10—N3—C9	121.37 (14)	C12—C13—H13	126.4
C14—N4—C11	108.84 (13)	N4—C14—C13	108.77 (13)
C14—N4—H400	125.6	N4—C14—H14	125.6
C11—N4—H400	125.6	C13—C14—H14	125.6
N1—C1—C2	108.79 (16)	O1—C15—O2	124.51 (16)
N1—C1—H1	125.6	O1—C15—C7	125.64 (16)
C2—C1—H1	125.6	O2—C15—C7	109.85 (13)
C1—C2—C3	106.93 (15)	O2—C16—C17	110.00 (14)
C1—C2—H2	126.5	O2—C16—H16A	109.7
C3—C2—H2	126.5	C17—C16—H16A	109.7
C4—C3—C2	108.02 (16)	O2—C16—H16B	109.7
C4—C3—H3	126.0	C17—C16—H16B	109.7
C2—C3—H3	126.0	H16A—C16—H16B	108.2
N1—C4—C3	107.07 (14)	C16—C17—H17A	109.5
N1—C4—C5	123.16 (16)	C16—C17—H17B	109.5
C3—C4—C5	129.77 (16)	H17A—C17—H17B	109.5
N2—C5—C4	120.53 (15)	C16—C17—H17C	109.5
N2—C5—H5	119.7	H17A—C17—H17C	109.5
C4—C5—H5	119.7	H17B—C17—H17C	109.5
N2—C6—C7	124.12 (15)	O3—C18—O4	124.90 (15)
N2—C6—S1	124.39 (13)	O3—C18—C8	121.72 (16)
C7—C6—S1	111.31 (11)	O4—C18—C8	113.35 (12)
C6—C7—C8	112.52 (15)	O4—C19—C20	107.18 (14)
C6—C7—C15	123.18 (14)	O4—C19—H19A	110.3
C8—C7—C15	124.26 (14)	C20—C19—H19A	110.3
C9—C8—C7	113.87 (15)	O4—C19—H19B	110.3
C9—C8—C18	119.05 (14)	C20—C19—H19B	110.3
C7—C8—C18	126.54 (14)	H19A—C19—H19B	108.5
C8—C9—N3	122.62 (15)	C19—C20—H20A	109.5
C8—C9—S1	111.38 (12)	C19—C20—H20B	109.5
N3—C9—S1	125.95 (13)	H20A—C20—H20B	109.5
N3—C10—C11	121.45 (15)	C19—C20—H20C	109.5
N3—C10—H10	119.3	H20A—C20—H20C	109.5
C11—C10—H10	119.3	H20B—C20—H20C	109.5
N4—C11—C12	107.92 (13)		
			
C4—N1—C1—C2	−0.1 (2)	C10—N3—C9—S1	−11.7 (2)
N1—C1—C2—C3	0.1 (2)	C6—S1—C9—C8	1.58 (13)
C1—C2—C3—C4	0.0 (2)	C6—S1—C9—N3	−175.84 (14)
C1—N1—C4—C3	0.08 (19)	C9—N3—C10—C11	178.43 (15)
C1—N1—C4—C5	−179.37 (15)	C14—N4—C11—C12	−0.75 (19)
C2—C3—C4—N1	−0.02 (19)	C14—N4—C11—C10	176.99 (15)
C2—C3—C4—C5	179.37 (16)	N3—C10—C11—N4	−6.6 (3)
C6—N2—C5—C4	−174.69 (14)	N3—C10—C11—C12	170.66 (17)
N1—C4—C5—N2	−2.4 (2)	N4—C11—C12—C13	0.7 (2)
C3—C4—C5—N2	178.28 (16)	C10—C11—C12—C13	−176.88 (17)
C5—N2—C6—C7	172.80 (15)	C11—C12—C13—C14	−0.4 (2)
C5—N2—C6—S1	−1.8 (2)	C11—N4—C14—C13	0.5 (2)
C9—S1—C6—N2	173.29 (13)	C12—C13—C14—N4	−0.1 (2)
C9—S1—C6—C7	−1.94 (12)	C16—O2—C15—O1	3.6 (2)
N2—C6—C7—C8	−173.42 (14)	C16—O2—C15—C7	−177.18 (12)
S1—C6—C7—C8	1.82 (16)	C6—C7—C15—O1	−17.3 (2)
N2—C6—C7—C15	4.4 (2)	C8—C7—C15—O1	160.28 (15)
S1—C6—C7—C15	179.65 (12)	C6—C7—C15—O2	163.54 (14)
C6—C7—C8—C9	−0.63 (19)	C8—C7—C15—O2	−18.9 (2)
C15—C7—C8—C9	−178.43 (14)	C15—O2—C16—C17	87.68 (18)
C6—C7—C8—C18	170.77 (15)	C19—O4—C18—O3	7.5 (2)
C15—C7—C8—C18	−7.0 (2)	C19—O4—C18—C8	−174.19 (13)
C7—C8—C9—N3	176.67 (14)	C9—C8—C18—O3	81.61 (19)
C18—C8—C9—N3	4.6 (2)	C7—C8—C18—O3	−89.4 (2)
C7—C8—C9—S1	−0.86 (17)	C9—C8—C18—O4	−96.74 (18)
C18—C8—C9—S1	−172.96 (11)	C7—C8—C18—O4	92.26 (18)
C10—N3—C9—C8	171.18 (16)	C18—O4—C19—C20	163.89 (14)

### Hydrogen-bond geometry (Å, °)

**Table:**

*D*—H···*A*	*D*—H	H···*A*	*D*···*A*	*D*—H···*A*
N1—H100···O3^i^	0.88	2.37	3.040 (2)	133
N4—H400···O1^ii^	0.88	2.47	3.174 (2)	138
N4—H400···N2^ii^	0.88	2.59	3.2136 (19)	128
C3—H3···N4^iii^	0.95	2.56	3.441 (2)	155
C13—H13···O3^iv^	0.95	2.51	3.126 (2)	123

Symmetry codes: (i) *x*+1/2, −*y*+3/2, *z*; (ii) *x*−1/2, −*y*+3/2, *z*; (iii) −*x*, −*y*+2, *z*−1/2; (iv) −*x*−1/2, *y*+1/2, *z*−1/2.

## References

[bb1] Bruker (2003). *SMART.* Bruker AXS Inc., Madison, Wisconsin, USA.

[bb2] Bruker (2004). *SAINT.* Bruker AXS Inc., Madison, Wisconsin, USA.

[bb3] Dufresne, S., Bourgeaux, M. & Skene, W. G. (2006). *Acta Cryst.* E**62**, o5602–o5604.

[bb4] Dufresne, S., Bourgeaux, M. & Skene, W. G. (2007). *J. Mater. Chem.* **17**, 1–13.

[bb5] Dufresne, S. & Skene, W. G. (2010*a*). *Acta Cryst.* E**66**, o3027. 10.1107/S1600536810043746 PMC3009046 21589183

[bb6] Dufresne, S. & Skene, W. G. (2010*b*). *Acta Cryst.* E**66**, o3221. 10.1107/S1600536810046775 PMC3011617 21589512

[bb7] Dufresne, S. & Skene, W. G. (2011). *J. Phys. Org. Chem* DOI 10.1002/poc.1894.

[bb8] Farrugia, L. J. (1997). *J. Appl. Cryst.* **30**, 565.

[bb14] Flack, H. D. (1983). *Acta Cryst.* A**39**, 876–881.

[bb9] Marris, T. (2004). *UdMX* Université de Montréal, Québec, Canada.

[bb10] Ruban, G. & Zobel, D. (1975). *Acta Cryst.* B**31**, 2632–2634.

[bb11] Sheldrick, G. M. (1996). *SADABS* University of Göttingen, Germany.

[bb12] Sheldrick, G. M. (2008). *Acta Cryst.* A**64**, 112–122. 10.1107/S0108767307043930 18156677

[bb13] Zobel, D. & Ruban, G. (1978). *Acta Cryst.* B**34**, 1652–1657.

